# Trends in COVID-19 vaccine administration across visit types in a safety net pediatric practice during the first year of authorization

**DOI:** 10.3389/fped.2023.1227115

**Published:** 2023-11-06

**Authors:** Grace W. Ryan, Melissa Goulding, Angela L. Beeler, Beverly L. Nazarian, Lori Pbert, Milagros C. Rosal, Stephenie C. Lemon

**Affiliations:** ^1^ Division of Preventive and Behavioral Medicine, Department of Population and Quantitative Health Sciences, University of Massachusetts Chan Medical School, Worcester, MA, United States; ^2^ Department of Pediatrics, UMass Memorial Health, Worcester, MA, United States; ^3^ Department of Pediatrics, UMass Chan Medical School, Worcester, MA, United States

**Keywords:** COVID-19 vaccination, electronic health record data, pediatrics, safety-net clinic, vaccine administration

## Abstract

We explored patterns of COVID-19 vaccination across pediatric visit types using electronic health record data from 7/1/2021 through 7/25/2022 in a pediatric safety-net clinic. We generated frequencies and descriptive statistics for patient demographic and vaccine administration variables. Analyses were stratified into age subgroups of 5-to-11-year-olds and 12- to-17-year-olds. 1,409 children received at least one dose of the COVID-19 vaccine and 2,197 doses were administered in this first year of vaccine delivery. Most vaccines given were first doses in the series (45%), followed by second doses (38%), and then booster doses (17%). First doses tended to be given at well-child (42%) or nurse visits (48%), while second doses were almost entirely given at nurse visits (87%) and booster doses at well-child visits (58%). Efforts to optimize COVID-19 vaccination could leverage clinic workflow systems to provide reminder prompts for vaccination for scheduling future doses and identify strategies to facilitate vaccination at non-well child visits, particularly for booster doses.

## 1. Introduction

COVID-19 vaccination prevents severe disease and hospitalization in children and adolescents (1). While the COVID-19 vaccines have been available to adolescents (ages 12–15) since Spring 2021, and to 5- to 11-year-olds since Fall 2021 (2), vaccination rates for these populations have stalled and as of December 2022 only 32% of 5- to 11-year-olds and 61% of 12- to 17-year-olds had completed their primary series (3). As COVID-19 reaches endemic levels and additional booster shots may be recommended, it is critical to establish long-term strategies for vaccinating pediatric populations.

Pediatric practices are the most likely delivery site for COVID-19 vaccines among children moving forward. Across multiple studies, parents have reported their pediatricians' offices as the most desirable location to get their children vaccinated (4, 5) and research on other vaccines indicates that pediatricians are trusted messengers and play a critical role in vaccine promotion (6). However, in comparison to other pediatric vaccines, COVID-19 vaccine delivery within pediatric practice presents unique challenges. These include requiring a 15-min post-vaccination waiting period, multiple doses with specific time intervals, and in some cases more rigorous storage requirements than other pediatric vaccines (e.g., the Pfizer-BioNTech vaccine requires storage at negative 130 to negative 76 degrees Fahrenheit). It is important to note that due to these logistical challenges (i.e., constantly changing guidelines; time required to reconstitute vaccines), not all pediatric settings are able to offer these vaccines (7). A national analysis conducted between November 2021 and April 2022 found that only 30% of US counties had a pediatric provider offering COVID-19 vaccines (8).

Despite the clear role of pediatric practices in delivering vaccinations, little is known about COVID-19 administration patterns in this setting. Gaining a better understanding of vaccine delivery in the pediatric practice setting could support future vaccine promotion efforts. With other vaccines, for example, successful strategies for increasing uptake include recommending and offering vaccines at all types of visits (9), not just well-child visits, and offering nurse or immunization-only visits to ease scheduling burden for parents. To begin identifying potential strategies to optimize COVID-19 vaccine delivery, we analyzed vaccine administration data in a safety net pediatric clinic within a system affiliated with a medical center in Central Massachusetts to explore trends in uptake and delivery patterns during the first year of COVID-19 vaccine delivery.

## 2. Methods

Patient-level vaccine administration data from a pediatric clinic within the UMass Memorial Health system was obtained from electronic health records (EHR) (Epic). The clinic began administering COVID-19 vaccines beginning 7/13/2021, and thus our data ranges from 7/13/2021 through 7/25/2022. The clinic exclusively offers the Pfizer-BioNTech vaccine. On an average day the clinic is staffed by 4 FTE (full time equivalent) of physician time, 2 FTE nurse practitioners, and 3 FTE nursing staff. Overall, the clinic has approximately 18,000 pediatric visits per year. Most of their patient population is covered by Medicaid (60%), 40% identifies as Hispanic/Latino and 10% prefers a language other than English.

EHR data on all patients between ages 5 and 17 who received any COVID-19 vaccination during the time period of interest included: date of vaccine administration, dose in the series (first, second, or booster dose), type of visit at which vaccine administration occurred (i.e., well child; follow-up; nurse-only; or sick visit), patient sex, age, race/ethnicity and language. We generated frequencies and descriptive statistics for all variables of interest. We used ***χ*^2^** tests to assess differences in demographics across types of visits and to explore differences in types of visits for different doses in the series. All analyses were stratified into age subgroups of 5- to 11-year-olds and 12- to 17-year-olds. Vaccine administration data were aggregated and graphed by month to explore trends over time. Additionally, we used data from the Massachusetts Department of Public Health on monthly COVID-19 case counts to assess trends in vaccine administration during surges of the pandemic. This study received a not human subjects' determination from the University of Massachusetts Chan Medical School Institutional Review Board.

## 3. Results

Overall, 1,409 children were vaccinated in the clinic from July 13, 2021 to July 25, 2022. A little over half (55%) of those vaccinated were in the 5–11 age group. Child demographic characteristics by the visit type of the first COVID-19 vaccine dose that they received in the clinic are shown in Table 1. We observed significant associations between age category (*p* < 0.001) and race/ethnicity (*p* = 0.02) by visit type. In this first year of vaccine administration a total of 2,197 doses were administered to the 1,409 unique patients. In terms of dose in the series, the largest percentage of those administered were first doses (45%), followed by second doses (38%) and booster doses (17%). Of the patients who received a first dose at this clinic, 72% returned for their second dose there (70% in 5- to 11-year-olds and 75% in 12- to 17-year-olds).

**Table 1:** Patient demographics by type of visit for first dose of COVID-19 vaccine series at a pediatric safety-net clinic, Worcester MA, July 2021 to July 2022 (*N* = 1,409).

	Overall	Well child visit	Nurse visit	Follow-up Visit	Sick visit	*χ*^2^ *p*-value
Age						
5–11 years	55.2%	46.6%	67.1%	46.7%	40.6%	
12–17 years	44.8%	53.3%	32.9%	53.4%	59.4%	*p* < 0.001
Sex						
Female	50.2%	49.1%	49.6%	55.6%	59.4%	
Male	49.8%	50.9%	50.4	44.4%	40.6%	*p* = 0.31
Language						
English	83.6%	83.5%	85.0%	80.0%	76.6%	
Spanish	10.4%	9.6%	10.4%	12.2%	17.2%	
Portuguese	2.0%	2.9%	0.8%	3.3%	1.6%	
Other	4.0%	4.0%	3.8%	4.4%	4.7%	*p* = 0.20
Race/Ethnicity						
Non-Hispanic White	28.0%	28.1%	28.8%	25.6%	23.4%	
Non-Hispanic Black or African American	19.7%	19.1%	22.1%	14.4%	9.4%	
Non-Hispanic Asian	7.1%	6.3%	7.9%	6.7%	7.8%	
Non-Hispanic other (including American Indian, Alaska Native, Native Hawaiian, Pacific Islander)	5.5%	4.5%	6.9%	2.2%	7.8%	
Hispanic	37.1%	38.9%	32.0%	47.8%	51.6%	
Missing	2.6%	3.1%	2.3%	3.3%	0.0%	*p* = 0.02

^a^ Totals may not add to 100% due to rounding.

**Figure 1:**
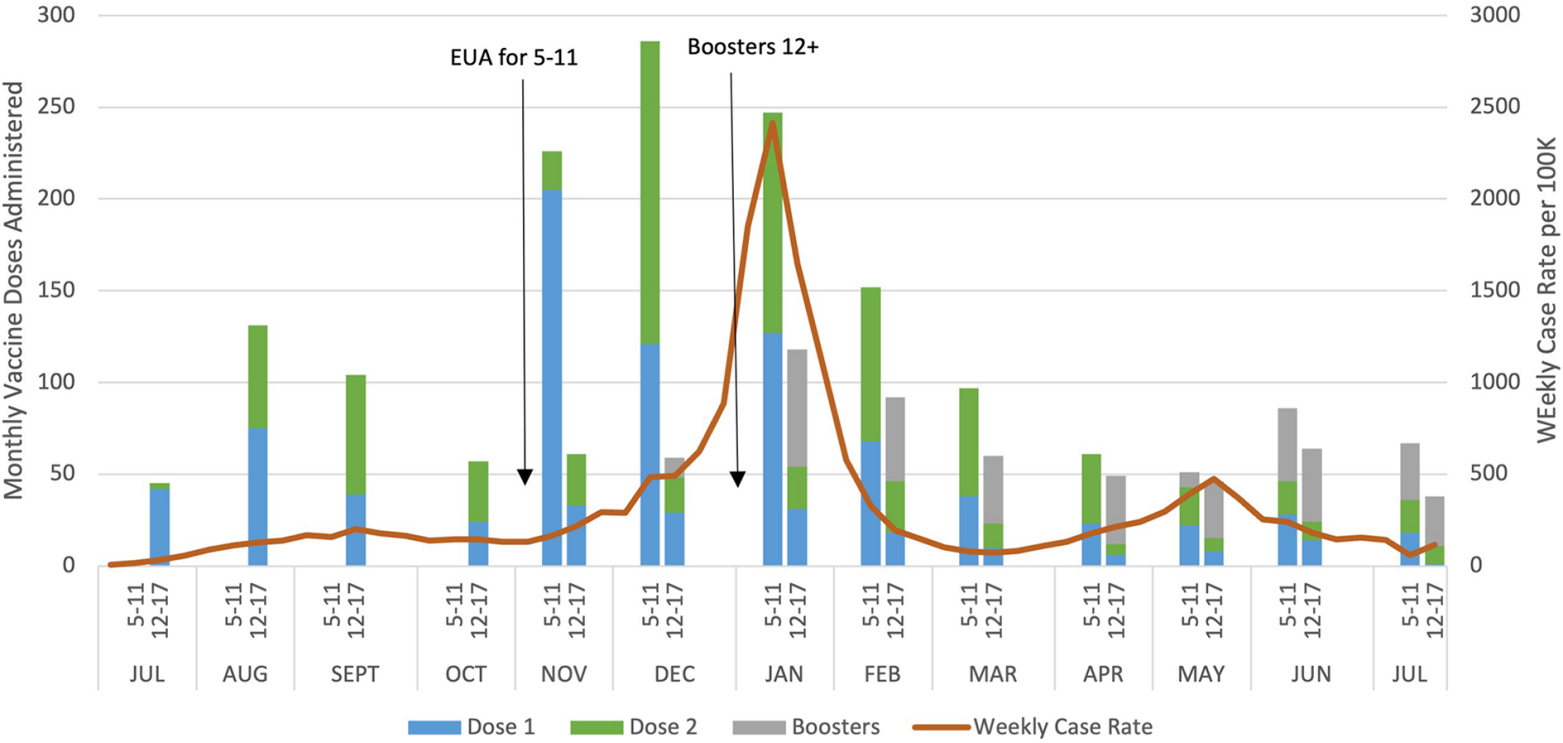
COVID-19 vaccine administration in a pediatric safety-net clinic, Worcester, MA and Worcester County weekly case rates July 2021 to July 2022.

We also explored at which types of visits doses were typically administered, and ***χ*^2^** test indicated a statistically significant association between dose and visit type. First doses of the series tended to be given at well-child visits (42.4%) or nurse visits (48.8%), while second doses were almost entirely given at nurse visits (87.9%) and booster doses at well-child visits (58.9%) (Table 2). Finally, we graphed monthly uptake of the vaccine to assess the relationship between uptake and local COVID-19 cases. Figure 1 displays doses of the COVID-19 vaccine administered by age group and month. For the 5–11-year-old age group, most doses were administered during the 3 months after Emergency Use Authorization (EUA) approval (November 2021 through January 2022) with most doses being the first in the series. For the 12–17-year-old age group, the most doses were administered in August 2021 and these doses were split between the first and second in the series. Figure 1 also depicts weekly case rates for Worcester County, where most of the clinic's patients reside, and highlights the spike caused by the Omicron variant with cases beginning to rise in November 2021, and ultimately peaking to nearly 2,500 cases per 100,000 residents in January 2022.

**Table 2:** COVID-19 vaccine doses administration by Age group and type of visit from July 2021 to July 2022 in a pediatric safety-net clinic, Worcester, MA (*N* = 2,197).

	Dose 1 (*n* = 980)	Dose 2 (*n* = 845)	Dose 3+ (*n* = 372)	Overall Pearson χ^2^
Follow-up visit	5.2%	3.1%	11.6%	
Nurse visit	48.8%	87.9%	22.0%	
Well child visit	42.4%	7.1%	58.9%	
Sick visit	3.7%	1.9%	7.5%	
				*p* < 0.001

## 4. Discussion

Examining trends in COVID-19 vaccine administration at a pediatric practice in the first-year post-approval for 12- to 17-year-olds and 5- to 11-year-olds, we gained important insight into when these vaccines are being administered and how that might be influenced by contextual factors. These findings have implications for COVID-19 vaccine delivery going forward, and also more broadly for other seasonal or multi-dose vaccines (e.g., HPV, influenza). With COVID-19 vaccination rates among pediatric populations lagging and the likelihood that further booster doses may be needed (10, 11), it is critical to improve vaccine delivery to ensure the prevention of excess morbidity and mortality from COVID-19 infections.

In exploring at which visit types COVID-19 vaccines were administered, an interesting pattern arose which gives context to who may be initiating vaccination. We found that first doses were often administered at nurse or well child visits, suggesting that this dose may be parent and provider driven as parents would initiate a nurse visit for the purpose of vaccination and providers would recommend vaccination at well visits. Second doses primarily occurred at nurse visits again implying parent and provider initiation as nurse visits for a second dose could be booked independently by the parent if the first dose was received elsewhere or based on provider's request to return for the second dose. In contrast, our data implied booster doses were mainly provider driven. We found that booster doses were most common at well-child visits but also administered more than the other doses at follow-up and sick visits. The administration of vaccines at these visits may indicate that providers in this clinic are trying to take advantage of all opportunities to vaccinate children, rather than waiting for preventive care or parent-initiated visits. This strategy of reducing missed opportunities by taking better advantage of acute care visits (i.e sick visits or follow up visits) has been shown to increase rates for other vaccines (12, 13) and should be promoted for use with COVID-19 vaccination. Given that the primary contraindications for COVID-19 vaccination are allergic reaction or active COVID-19 infection, there are many instances in which children may be in their pediatric practice and eligible for vaccination. Strategies like integrating EHR pop-ups to check vaccine status at all visit types (14–16) may help to further encourage providers to take advantage of all visits either to vaccinate or to schedule a return visit for vaccination. Additionally, offering nurse-only visits which parents can schedule specifically for vaccination and potentially increasing nursing staff capacity to accommodate these appointments may be important strategies in supporting pediatric practices' capacity to vaccinate.

We also were able to discern trends in administration based on contextual factors. For example, we saw increases in COVID-19 vaccine doses administered prior to the beginning of the school year and in the weeks during this county's Omicron surge. August is often a popular time to target vaccine promotion efforts due to the influx of children coming into clinics for well child and sports' physicals, going forward COVID-19 vaccination should also be promoted during this time. Moreover, while ideally continued vaccination will prevent future COVID-19 surges like the one experienced due to Omicron, knowing the patterns of the virus allows us to predict optimal timing for vaccination. Similar to influenza and other respiratory viruses, winter surges seem common with COVID-19, therefore messaging should focus on getting vaccinated prior to these potential surges, rather than during. Additionally, pediatric practices can and should prepare to increase capacity for vaccine administration during these times.

Beyond understanding these patterns, our results further support the need to focus on pediatric practice clinics as the ideal setting for pediatric COVID-19 vaccination. There is extensive research showing that parents would prefer to get their child vaccinated in their own pediatricians' offices (4, 5). In our study, we found that 72% of children who received their first dose at the clinic returned for their second dose, suggesting that parents felt comfortable or appreciated the convenience of getting their child vaccinated at their pediatricians' office, as they do with most other routine vaccines. Furthermore, the percentage of 5- to 11-year-olds who returned for their second dose at this clinic (70%) is considerably higher than the rate for the entire state of Massachusetts (53%) (17). While this difference may be due to differential uptake and vaccine perceptions across the state, one possible explanation is that it could be due to more positive vaccine experiences within pediatric clinics as compared to experiences in alternative vaccine settings (e.g., community-settings, pharmacies) (18, 19). Although there is a push for alternative vaccine settings, it seems clear that for the pediatric population, the focus needs to be on supporting pediatricians in their ability to administer COVID-19 vaccines within their practices.

### 4.1. Limitations

There are several limitations that should be acknowledged in interpreting our results. First, these results are limited to a single clinic within an academic medical center, and not representative of the experience of all clinics, especially clinics that are community-based, in other geographic areas, or that lack the capacity to offer the COVID-19 vaccines. Moreover, vaccine rollout varied state to state and in Massachusetts, mass vaccination sites were prioritized over individual clinics, which could have affected uptake. Furthermore, we do not have access to these patients' full medical records, therefore it is possible that they received doses outside of this clinic. This would be especially true for patients between ages 12 and 17 as the clinic began offering the vaccine three months after EUA for this age group. Likewise, this makes it impossible in this analysis to estimate prevalence of vaccination receipt within the clinic. Despite these limitations, our results offer insight into challenges of pediatric COVID-19 vaccine delivery and highlight potential strategies for improving vaccination in this age group.

### 4.2. Conclusions

This analysis allowed the exploration of patterns in COVID-19 vaccination and began to identify opportunities to improve vaccine delivery. Other clinical settings may see different vaccination trends and a similar exercise could help them identify opportunities to improve COVID-19 vaccine delivery. Future work in this area could prioritize optimizing COVID-19 vaccine delivery within pediatric practices by leveraging clinic workflow systems to provide reminder prompts for vaccination for scheduling future doses or identifying strategies to facilitate vaccination at non-well child visits. Ultimately, this analysis shed light on the ways in which clinic-level data can be used to understand these trends and the utility in exploring vaccine administration data in this way.

## Data Availability

The data analyzed in this study is subject to the following licenses/restrictions: We used electronic health record data proprietary to our health system that contains PHI therefore we are unable to share this data. Requests to access these datasets should be directed to grace.ryan1@umassmed.edu.
